# Sequential Induction of Effector Function, Tissue Migration and Cell Death during Polyclonal Activation of Mouse Regulatory T-Cells

**DOI:** 10.1371/journal.pone.0050080

**Published:** 2012-11-30

**Authors:** Daniela Langenhorst, Tea Gogishvili, Eliana Ribechini, Susanne Kneitz, Kirsty McPherson, Manfred B. Lutz, Thomas Hünig

**Affiliations:** 1 Institute for Virology and Immunobiology, University of Würzburg, Würzburg, Germany; 2 Interdisciplinary Centre for Clinical Research (IZKF), University of Würzburg, Würzburg, Germany; 3 Department of Microbiology and Immunology, The University of Melbourne, Melbourne, Australia; Julius-Maximilians-Universität Würzburg, Germany

## Abstract

The ability of CD4^+^Foxp3^+^ regulatory T-cells (Treg) to produce interleukin (IL)-10 is important for the limitation of inflammation at environmental interfaces like colon or lung. Under steady state conditions, however, few Tregs produce IL-10 *ex vivo*. To investigate the origin and fate of IL-10 producing Tregs we used a superagonistic mouse anti-mouse CD28 mAb (CD28SA) for polyclonal *in vivo* stimulation of Tregs, which not only led to their numeric expansion but also to a dramatic increase in IL-10 production. IL-10 secreting Tregs strongly upregulated surface receptors associated with suppressive function as compared to non-producing Tregs. Furthermore, polyclonally expanding Tregs shifted their migration receptor pattern after activation from a CCR7^+^CCR5^−^ lymph node-seeking to a CCR7^−^CCR5^+^ inflammation-seeking phenotype, explaining the preferential recruitment of IL-10 producers to sites of ongoing immune responses. Finally, we observed that IL-10 producing Tregs from CD28SA stimulated mice were more apoptosis-prone *in vitro* than their IL-10 negative counterparts. These findings support a model where prolonged activation of Tregs results in terminal differentiation towards an IL-10 producing effector phenotype associated with a limited lifespan, implicating built-in termination of immunosuppression.

## Introduction

CD4^+^Foxp3^+^ regulatory T-cells (Tregs) are essential for the maintenance of immunological homeostasis and self tolerance [1]. They control unwanted immune responses by a number of distinct mechanisms, which differentially contribute to suppression depending on the experimental settings studied [2]. One key element in the regulation and execution of Treg activity is interleukin (IL)-2. Thus, IL-2 produced by “conventional” T-cells (Tconvs) drives Treg expansion and boosts regulatory function, including production of the suppressive cytokine IL-10 [3]. At the same time, Tregs act as an “IL-2 sink” [4] since they do not produce IL-2 themselves [5] but consume IL-2 produced by Tconvs.

IL-10 produced by several innate and adaptive immune cells is a key immunosuppressive and anti-inflammatory cytokine as illustrated by the spontaneous lethal inflammatory bowel disease of IL-10-deficient mice [6]. IL-10-mediated control of immune homeostasis has been associated with several CD4^+^ T-cell subsets defined by their anatomical origin or mode of generation like Foxp3^−^IL-10^+^ Tr1 (T regulatory type-1), Tr1-like cells and Foxp3^+^ Tregs, the latter being the subject of the present paper. Foxp3^+^ Tregs can develop in the thymus or in the periphery from naïve CD4^+^ T-cell precursors [7]. *In vitro* studies failed to show a requirement for IL-10 in the inhibition of T-cell responses [8]. In contrast, Foxp3^+^ Treg derived IL-10 contributes to immune regulation *in vivo*, although not in all settings studied [2]. Selective IL-10 deficiency in Tregs results in spontaneous colitis and inflammation in skin and lung [9].

To locally suppress unwanted immune responses and inflammation, Tregs must migrate to lymphoid organs and inflamed tissues [10]. Homing and trafficking are controlled by adhesion molecules and chemokine receptors [11]. Consequently naïve Tregs express CD62L and CCR7, allowing them to recirculate through lymphoid tissues. Upon activation, Tregs upregulate inflammatory chemokine receptors (CCR2, CCR5, CCR6, CXCR3), selectin ligands (E- and P-selectin ligand) and integrins (CD103), directing them to inflamed sites [12], [13]. Accordingly, IL-10 producing Tregs are mainly found at sites of ongoing immune responses [7].

In a healthy immune system, the balance between Tregs and Tconvs is controlled by mechanisms which mutually control survival and proliferation of both types of T-cells, e.g., even in an apparently resting immune system, the survival of Tregs in the periphery is controlled by common gamma chain cytokines (mainly IL-2) derived from Tconvs [14], and by costimulatory signals via CD28 [15]. During an immune response, Treg expansion follows that of Tconvs, and is driven by antigen recognition and by excess IL-2 produced by the activated conventional CD4^+^ T-cells. Both populations of T-cells clonally contract once the antigen has been eliminated [16].

In contrast to clonal expansion of rare Treg clones, synchronous activation of a large number of Tregs allows for a more comprehensive characterisation of their phenotype, function, and migrational behaviour. In order to follow such a synchronous wave of polyclonal Treg activation *in vivo*, we have used stimulation with superagonistic mouse anti-mouse CD28 mAb (CD28SA). We and others have previously shown that such mAb are effective expanders and activators of Tregs in rats [17], [18] and mice [19], [20], [21], allowing interference with multiple model diseases of autoimmunity, graft rejection and inflammation [22].

CD28SA differ from conventional CD28-specific antibodies by their ability to activate T-cells without T-cell receptor (TCR) ligation by amplifying weak or “tonic” TCR signals [23], [24]. *In vitro* and *in vivo*, they act on both Tconvs and Tregs; the IL-2 produced by Tconvs in response to CD28SA stimulation is immediately utilized by Tregs [19], which respond with expansion and functional activation including IL-10 production [17], [18], [25]. This single wave of polyclonal Treg activation effectively controls the expression of effector functions and toxic cytokine release by Tconvs, which were also initially triggered [19], and is terminated by clonal contraction within two weeks after CD28SA application [19], [23].

Whereas it has long been accepted that IL-10 can be used by Tregs to suppress immune responses, there is only little known about the function and fate of Tregs with an IL-10 producing phenotype. In the present study we observed that IL-10 producing Tregs are derived from non-IL-10 producing Tregs. They are highly activated suppressor cells migrating to inflamed tissues. Furthermore the IL-10-producing phenotype is associated with an increased sensitivity to apoptosis. We suggest that IL-10 producing Tregs represent a fully activated stage of terminal differentiation with a limited life-span, which facilitates the return to immunological normality after clearance of an infection.

## Results

### CD28SA application induces IL-10 production in Tregs

**Figure 1:**
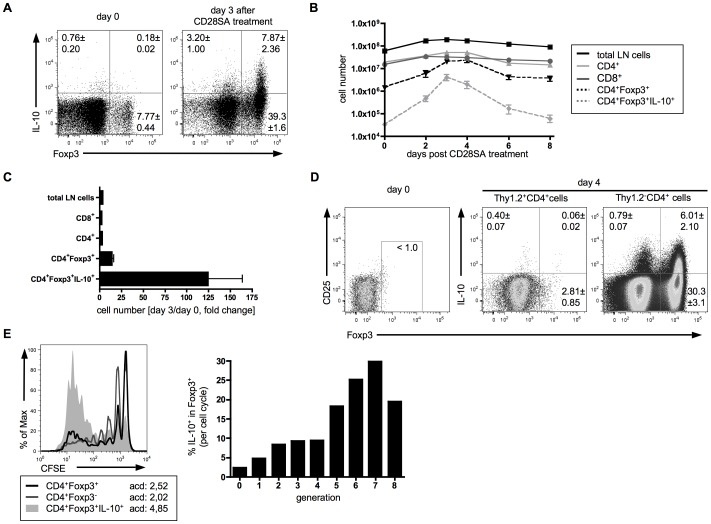
IL-10 and Foxp3 expression in CD4^+^ LN T-cells of untreated or CD28SA treated C57BL/6 mice. (**A**) IL-10 and Foxp3 expression in unstimulated mice and in mice 3 days after CD28SA treatment. (**B**) Kinetics of subsets defined by CD8, CD4, Foxp3 and IL-10 expression 0 to 8 days after CD28SA injection. (**C**) Fold change in cell numbers between day 0 and day 3 after stimulation. Graphs show means ± SD from 3 mice assayed individually and results are representative of three independent experiments. (**D**) Lack of conversion of CD4^+^ Tconv after CD28SA treatment. Sorted Thy1.2^+^CD4^+^ Foxp3^−^ cells from DEREG mice were transferred into naïve Thy1.1 mice one day before CD28SA injection. Three days later Thy1.2^+^ cells were analysed for Foxp3 and IL-10 expression. Graphs show means ± SD from 4 mice assayed individually and results are representative of two independent experiments. (**E**) Proliferative history and expression of Foxp3 and IL-10. CFSE-labeled CD4^+^ T-cells from Thy1.1 mice were transferred into naïve C57BL/6 mice one day before CD28SA injection. Three days later Thy1.1^+^ cells were analysed for proliferation, Foxp3 and IL-10 expression. Flow cytometric analysis of transferred T-cells with average numbers of cell division (acd) (left) and percentage of IL-10 producers within Foxp3^+^ cells per generation (right) are depicted. Data represent pooled cells from two mice, and results are representative of three independent experiments.

Tregs can be expanded and functionally activated by a single injection of the mouse anti-mouse superagonistic CD28 mAb D665 [19], [25]. We injected a saturating dose (200 µg/mouse, [19]) of CD28SA i.p. into C57BL/6 mice and followed Treg expansion and IL-10 production in lymph nodes. In unstimulated mice between 5 and 10% of the CD4^+^ T-cell population were Foxp3^+^ Tregs (**Fig. 1A**). Three days after CD28SA injection, their frequency increased up to six-fold and their absolute cell numbers up to 15-fold compared to control values (**Fig. 1B and 1C**). Whereas few IL-10 secreting Tregs are found in an apparently resting immune system, we observed a dramatic increase (125-fold) after administration of CD28SA (**Fig. 1A–1C**). The response to CD28SA peaked on day 3 to 4, and cell numbers went back to normal levels within 8 days (**Fig. 1B** and [19]). Transfer of Foxp3-depleted Thy1.2^+^ CD4^+^ T-cells from DEREG mice into Thy1.1 mice revealed that the increase in IL-10 production and Treg number three days after CD28SA treatment is mainly the result of expansion of pre-existing Treg cells, rather than conversion from CD4^+^ Tconv (**Fig. 1D**).

The high frequency of IL-10 producing Tregs in CD28SA treated mice allowed us to further investigate the origin and fate of these polyclonally stimulated Tregs. To examine the relationship between IL-10 production and the proliferative history of Tregs, we transferred CFSE-labeled CD4^+^ T-cells from Thy1.1 mice into congenic C57BL/6 mice. 24 hours later animals were treated with CD28SA, and after three days the progeny of transferred cells were analysed for the expression of Foxp3 and IL-10. Both conventional and Foxp3^+^ CD4^+^ T-cells proliferated, but Tregs proliferated more extensively than conventional CD4^+^ T-cells, most likely because of the counter-regulation exerted by Tregs (**Fig. 1E** and [22]). Importantly, IL-10 expression in Tregs was most prominent in cells that had undergone several cell divisions (**Fig. 1E**). Together with the kinetic data (**Fig. 1B**) this suggests that in response to *in vivo* stimulation with CD28SA, Tregs initially expand before they switch on IL-10 production.

### Suppressive capacity of IL-10^+^ CD28SA-activated Tregs

**Figure 2:**
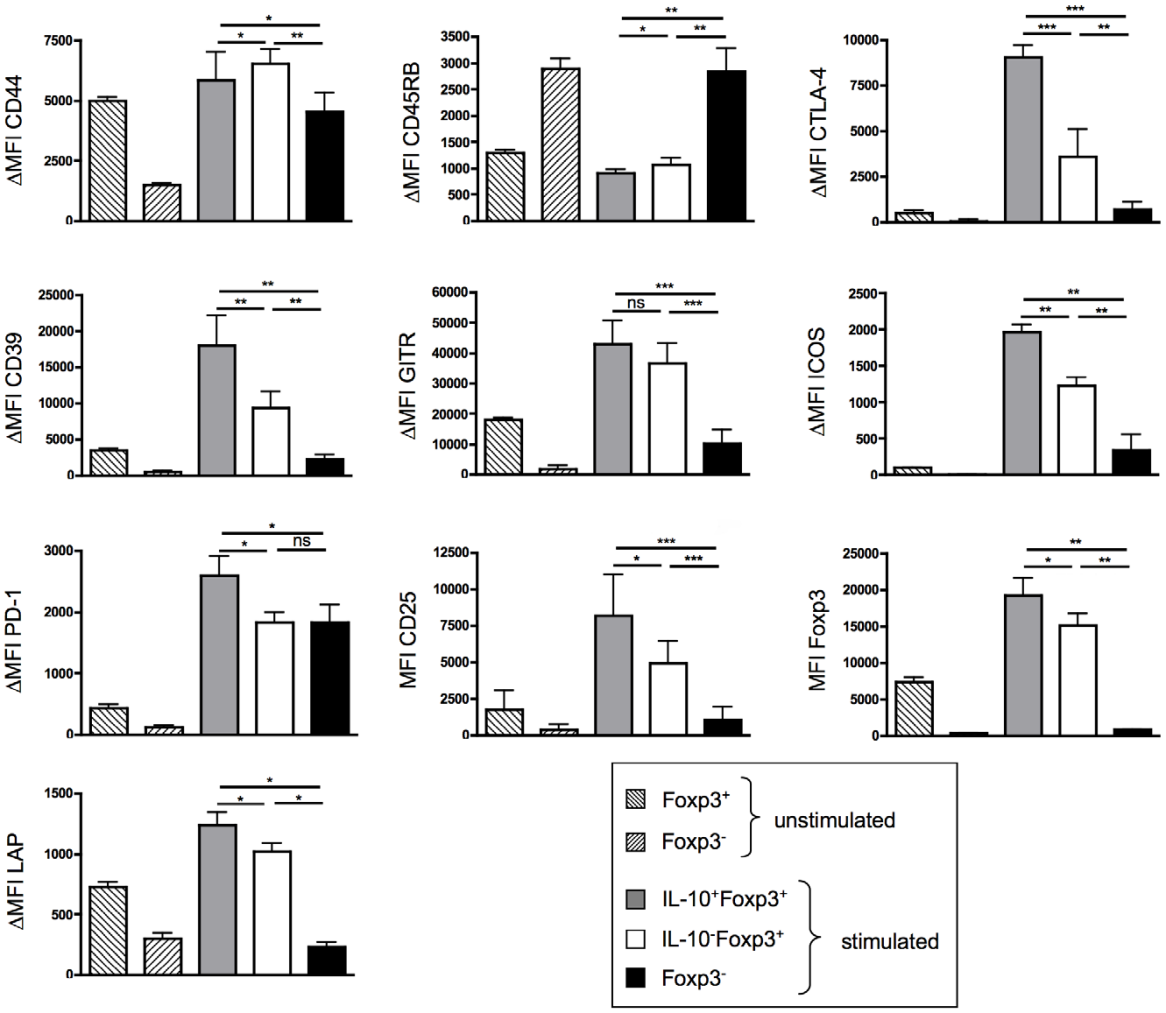
Phenotype of IL-10^+^ and IL-10^−^ Tregs 3 days after CD28SA treatment. ΔMFI (mean fluorescence intensity) ( = MFI(marker)−MFI(isotype)) of memory and Treg specific markers on unstimulated or on day 3 stimulated CD4^+^ T-cell subsets defined by Foxp3 and/or IL-10 expression. Graphs show means ± SD from 4–8 mice and results are representative of two independent experiments. * p<0.05, ** p<0.005, *** p<0.001, ns: no significant difference.

To further characterize the IL-10 producing and non-producing Treg subsets recovered three days after polyclonal *in vivo* activation with CD28SA, we phenotyped CD4^+^ T-cells by surface marker expression (**Fig. 2**). Unstimulated as well as stimulated Foxp3^+^ T-cells expressed a memory-like phenotype, i.e. in comparison to Tconvs (Foxp3^−^), they expressed high levels of CD44 and low levels of CD45RB. In addition, the functionally important Treg surface receptors CD25, CTLA-4, GITR and ICOS were further upregulated in Tregs from CD28SA stimulated mice. Interestingly, cell surface molecules associated with effector function of Tregs like CD39, PD-1, LAP, CD25 or CTLA-4 were expressed at even higher levels in IL-10 producing than in IL-10 negative Tregs, where upregulation was up to 20-fold (for CTLA-4) as compared to unstimulated Tregs (**Fig. 2**).

**Figure 3:**
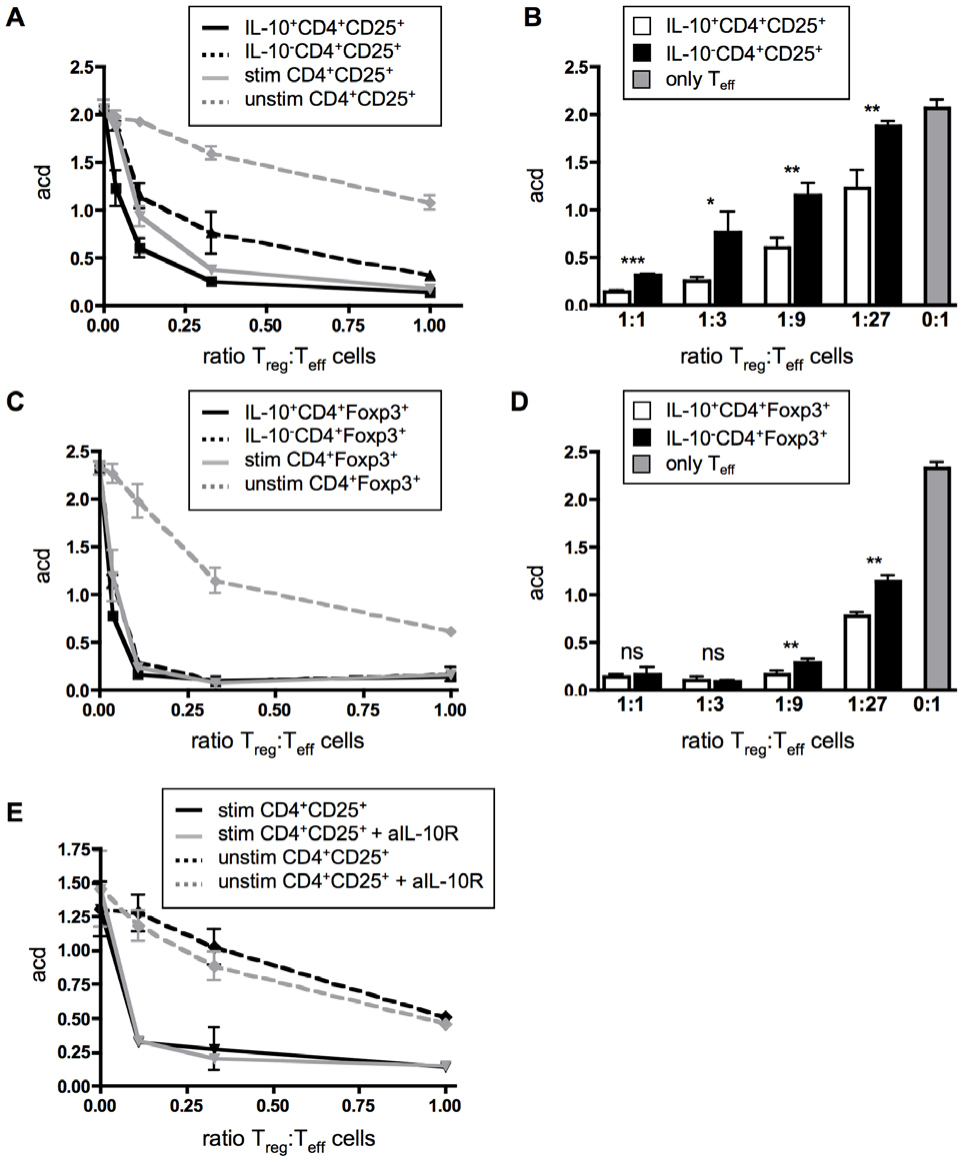
Suppressive activity of Treg subsets. (**A**) Isolated CD4^+^CD25^+^ Tregs from CD28SA treated mice were analysed for IL-10 production using IL-10 Secretion Assay. FACS separated IL-10^+^ (solid black line) and IL-10^−^ CD4^+^CD25^+^ Tregs (dashed black line) were incubated with CFSE-labeled naïve CD4^+^CD25^−^ effector T-cells (T_eff_), (ratio T_eff_∶ T_reg_ 1∶1 to 81∶1) and irradiated APC in the presence of anti-CD3 for 3 days. Stimulated total CD4^+^CD25^+^ Tregs (solid grey line) and CD4^+^CD25^+^ Tregs from untreated mice (dashed grey line) were used as controls. (**B**) Bar graph of IL-10^+^ (white bar) and IL-10^−^ CD4^+^CD25^+^ Tregs (black bar) with statistical analysis. (**C** and **D**) like (**A** and **B**) but with FACS sorted Foxp3^+^ of unstimulated and CD28SA stimulated DEREG mice. (**E**) Suppression is IL-10 independent. Blocking anti-IL-10R mAb (10 µg/ml, grey line) was added to cultures with stimulated (solid line) and unstimulated total CD4^+^CD25^+^ Tregs (dashed line). Graphs show average numbers of cell division (acd, mean ± SD) from 3 wells assayed individually and results are representative of three independent experiments. * p<0.05, ** p<0.005, *** p<0.001, ns: no significant difference.

To compare the suppressive activity of the *in vivo* expanded IL-10^+^ and IL-10^−^ Tregs from CD28SA stimulated mice, CD4^+^CD25^+^ cells were separated by FACS into IL-10^+^ and IL-10^−^ cells using an IL-10 capture assay. Alternatively IL-10^+^Foxp3^+^ and IL-10^−^Foxp3^+^ Tregs were FACS sorted from DEREG Foxp3-reporter mice [26]. These cell fractions were then cultured with purified CFSE-labeled CD4^+^CD25^−^ cells as responders, irradiated APCs and anti-CD3 as a proliferative stimulus. As controls, unseparated CD4^+^CD25^+^ or CD4^+^Foxp3^+^ T-cells from unstimulated and CD28SA stimulated mice were included. In comparison to Tregs from unstimulated mice, CD28SA stimulated Tregs showed a more than tenfold higher suppressive activity (**Fig. 3A, 3C** and [19]). Both IL-10^+^ and IL-10^−^ Tregs were highly suppressive, with a higher activity in the IL-10^+^ Treg population (**Fig. 3A–D**), in line with the elevated expression of Treg effector molecules by this subset (**Fig. 2**). As expected from the literature, failure of the anti-IL-10R blocking mAb to relieve inhibition confirmed that IL-10 itself is not an important factor in this *in vitro* setting (**Fig. 3E** and [7], [8]). Together, these results confirmed that *in vivo* pretreatment with the mouse CD28SA upregulates effector function of Tregs [19], [20], [21] and show that their high *in vitro* efficacy is associated with, but not dependent on, IL-10 production.

### CD28SA stimulated Tregs migrate to inflamed sites

**Figure 4:**
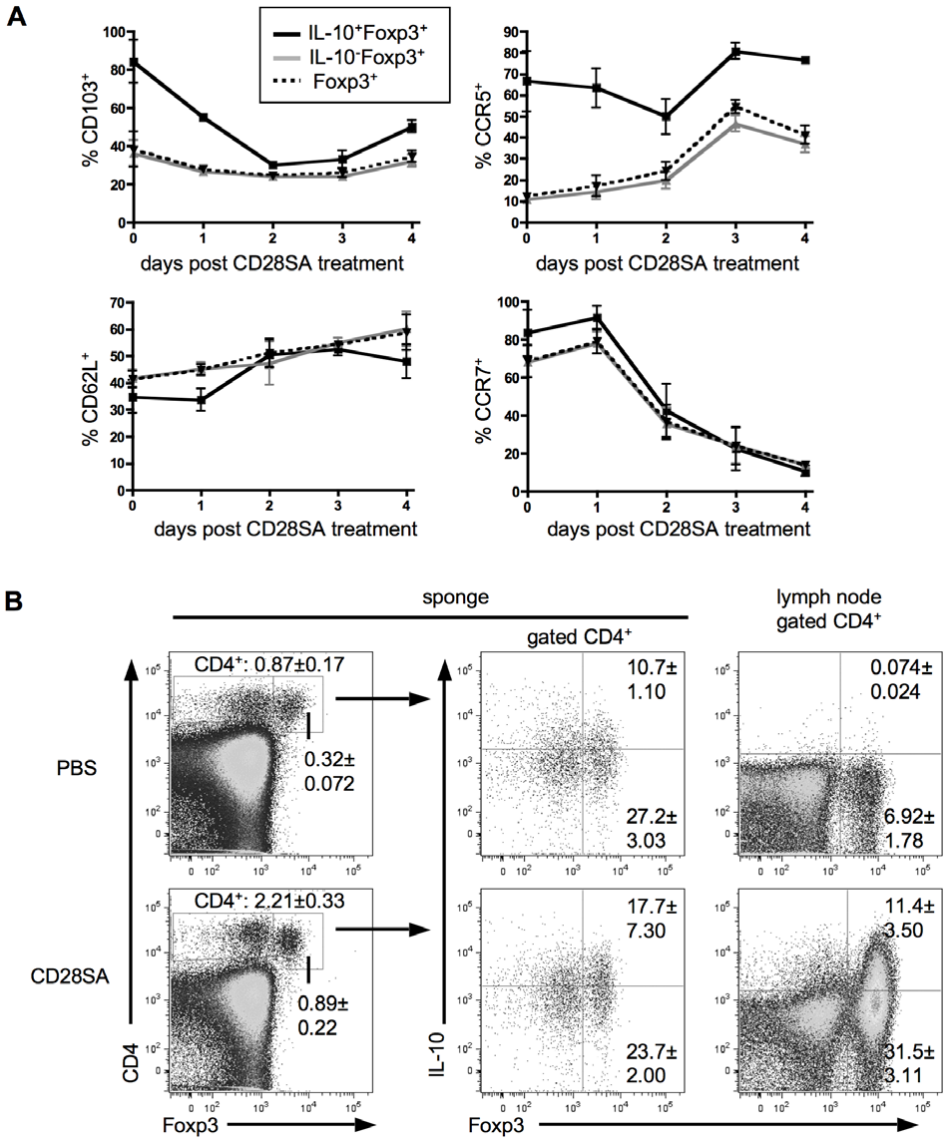
Migration potential of Tregs after CD28SA injection. (**A**) Flow cytometric analysis of homing related receptors in IL-10^+^Foxp3^+^ (black line), IL-10^−^Foxp3^+^ (grey line) and total Foxp3^+^ (dashed line) Tregs of mice 0 to 4 days after CD28SA treatment. (**B**) Migration of Tregs *in vivo*. A small piece of sponge containing heat-killed *Listeria monocytogenes* was placed under the skin of C57BL/6 mice. After 4 days mice were treated with CD28SA or PBS. On day 7 cells washed out of sponges were stained for CD4, IL-10 and Foxp3 and compared with CD4^+^ gated LN cells. Graphs show means ± SD from 3 mice assayed individually and results are representative of two independent experiments.

Treg migration to sites of ongoing immune responses is dependent on surface expression of chemokine receptors and adhesion molecules. Receptors like CCR5, CCR6 or CD103 direct cells to inflamed tissues, whereas CCR7 and CD62L are associated with lymph node homing [12], [13]. Here, we analyzed whether *in vivo* activated IL-10 secreting Tregs express the appropriate receptors for migration to inflamed tissues (**Fig. 4A** **and S1**). In lymph nodes of unstimulated mice about 35% of the total Treg population expressed CD103 and only about 10% expressed CCR5 or CCR6. In agreement with published data most of the Tregs expressed CCR7, and approximately 40% expressed CD62L [11]. After injection of CD28SA the pattern of migration receptors on Tregs shifted. Whereas the fraction of total Tregs expressing CD103 (minor change) and CD62L (increase from 40% to 60%) were not dramatically altered, there was a major change in the expression profile of CCR5, CCR6 and CCR7. The frequency of Tregs expressing the lymphoid homing receptor CCR7 was strongly decreased (from about 70% to 20–10% CCR7^+^ cells) after 3 to 4 days of CD28SA injection whereas those expressing CCR5 or CCR6, receptors for migration into inflamed tissues, were strongly increased (from about 10% to 50% CCR5^+^ or CCR6^+^ cells).

If IL-10 producing Tregs are activated, tissue-seeking descendants of non IL-10 producing cells in a resting immune system, a shift in migration markers should also be visible between these populations in unstimulated mice. Indeed comparing the small population of endogenous IL-10 producing with non-producing Tregs, we found that the former contained a higher proportion of tissue homing CD103^+^, CCR6^+^ and CCR5^+^ cells than non IL-10 secreting Tregs (**Fig. 4A** and data not shown). Also after *in vivo* stimulation with CD28SA, IL-10-producers contained a significantly higher fraction of CCR5^+^, CCR6^+^ and CD103^+^ cells than IL-10 non-producers (**Fig. S1**). Accordingly, the initial drop in the frequency of CD103^+^ among IL-10^+^Foxp3^+^ cells after CD28SA simulation (**Fig. 4A**) is likely due to a delay in CD103 expression following the induction of IL-10 expression. Regardless of IL-10 production however, CCR7 was downregulated in all Foxp3^+^ cells. From these data (**Fig. 1B, 1E** **,** **4A**) we hypothesized that upon activation, Tregs initially proliferate, downregulate CCR7, then start to secrete IL-10 and upregulate CCR5 and CCR6 to finally migrate into inflamed tissues.

To test this directly, a sponge implant containing heat-killed *Listeria monocytogenes* was inserted under the skin of C57BL/6 mice to induce inflammation. 4 days later mice were treated with CD28SA to expand Tregs. After one week sponges were removed and immigrated CD4^+^ T-cells were analysed for IL-10 and Foxp3 expression. Leukocytes recovered from sponges of untreated mice contained only few CD4^+^ T-cells within a monocyte/lymphocyte gate (0.87%±0.17), which were increased by 2–3 fold in the CD28SA-treated group (2.21%±0.33) (**Fig. 4B**). In both groups, about 40% of CD4^+^ T-cells were Foxp3^+^ and about one third of Foxp3^+^ cells produced IL-10, indicating that this phenotype is, indeed, inflammation seeking. In contrast to their enrichment at the site of inflammation, IL-10 producing Tregs were rare in the lymph nodes of PBS treated mice (about 1% of Foxp3^+^), but strongly increased after CD28SA-driven activation (about 25% of Foxp3^+^) (**Fig. 4B**). These data are best explained by preferential recruitment of IL-10 producers or at least IL-10 competent Tregs to the site of inflammation, resulting in a high local frequency of IL-10 producers even if only few are found in unstimulated secondary lymphoid tissue.

### IL-10 producing Tregs are not a stable subpopulation

The hypothesis that the steep increase in Tregs poised to release IL-10 observed after *in vivo* treatment with CD28SA is due to conversion from a Foxp3^+^IL-10 non-producer to a Foxp3^+^IL-10 producer phenotype was directly addressed both *in vitro* and *in vivo* by separating IL-10 producers from non-producers and following their fate.

**Figure 5:**
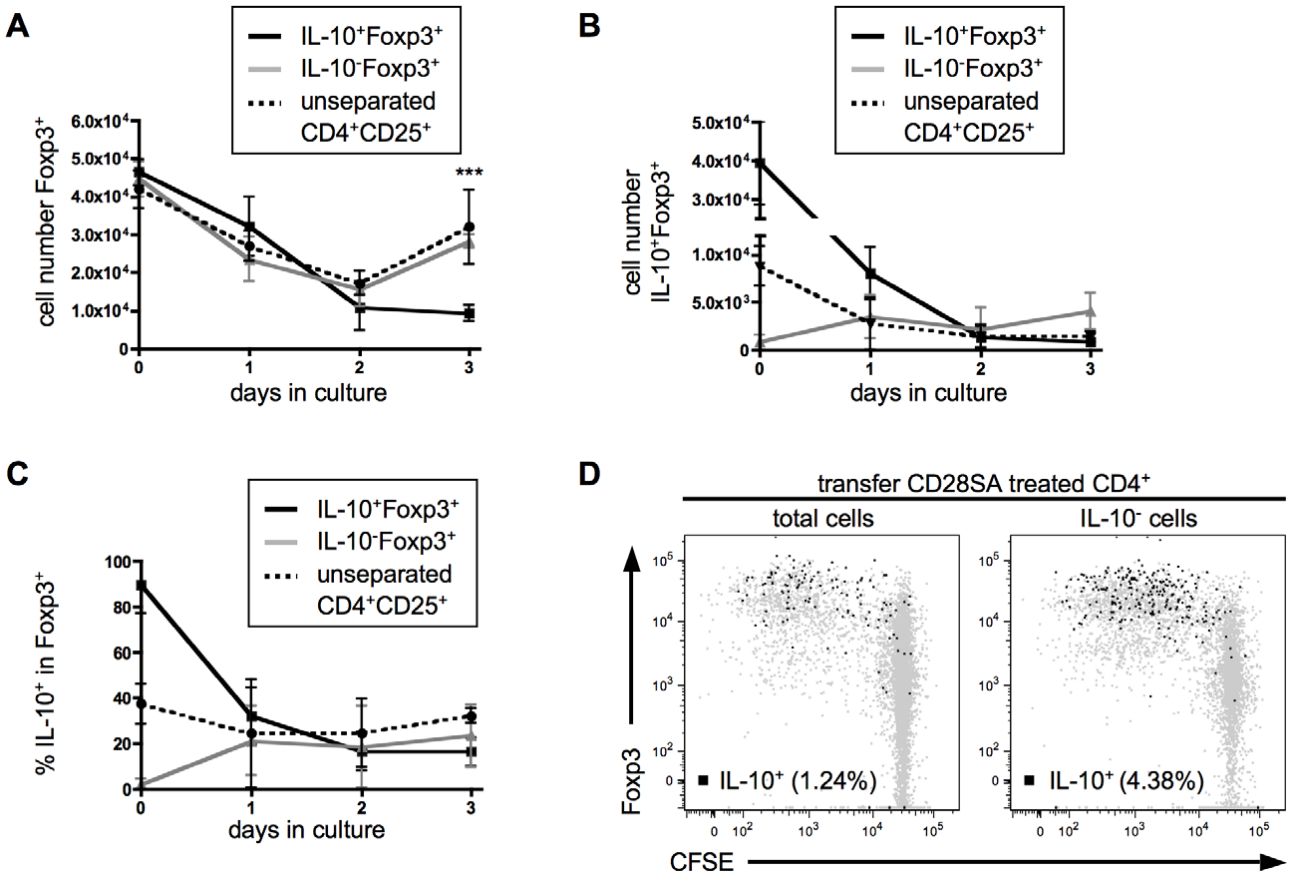
Instability of IL-10 producing phenotype *in vitro* and *in vivo*. (**A–C**) Phenotypic changes during *in vitro* proliferation. CD4^+^CD25^+^ Tregs of CD28SA treated DEREG mice were isolated and stimulated over night with anti-CD3, anti-CD28 and IL-2. Next day cells were stained for IL-10 and separated into IL-10^+^Foxp3^+^ (black line) and IL-10^−^Foxp3^+^ (grey line) via FACS Sorter. Isolated cells where cultured for 1 to 3 days with anti-CD3, anti-CD28, IL-2 and examined for cell numbers of Foxp3^+^ cells (**A**), of IL-10^+^Foxp3^+^ cells (**B**) and for percentage of IL-10 producers (**C**). As a control, unseparated CD4^+^CD25^+^ T-cells (dashed line) were used. Graphs show means ± SD of 3 individual experiments. *** p<0.001 compared IL-10^+^Foxp3^+^ to IL-10^−^Foxp3^+^ cells. (**D**) *In vivo* conversion. CFSE labeled total CD4^+^ T-cells or IL-10^−^CD4^+^ T-cells of Thy1.1 mice 3 days after CD28SA stimulation, were transferred into naïve C57BL/6 mice. One day after cell transfer recipient mice received CD28SA injection. 3 days later cells were re-isolated and analysed for proliferation, Foxp3 and IL-10 expression (black dots). Flow cytometric analysis of transferred T-cells with percentage of IL-10 producing cells is depicted. Graphs show means ± SD from 2–3 mice per group and results are representative of two independent experiments.

To obtain IL-10^+^Foxp3^+^ and IL-10^−^Foxp3^+^ Tregs of high purity, DEREG Foxp3-reporter mice [26] were treated with CD28SA three days earlier. CD4^+^ T-cells were cultured over night under optimized conditions of stimulation (anti-CD3/anti-CD28 costimulation and IL-2) increasing the recovery of IL-10 producers detected by a capture assay. Unseparated and FACS-separated cells were further cultured with anti-CD3/anti-CD28 plus IL-2 for three days, and analysed each day for the capacity to produce IL-10 after restimulation with PMA/Ionomycin. While initially, recovery of Foxp3 expressing cells declined in all three groups by about 50%, unseparated and IL-10-depleted Treg numbers stabilized on day 2 and then increased again (**Fig. 5A**). In contrast, viable Tregs derived from IL-10 producers declined further, resulting in only 20% recovery on day 3. Besides declining in number, however, the vast majority of initially IL-10-producing Tregs also ceased to produce this cytokine, resulting in a decline from 90% to 20% within 2 days (**Fig. 5B and 5C**). In contrast, a significant fraction (20%) of initially IL-10 negative Tregs became IL-10 producers within the first day of culture. Thus under optimal *in vitro* stimulation conditions, IL-10 producing Tregs declined both in number and in their capability to produce IL-10, whereas initially IL-10-negative Tregs survived better and gave rise to new IL-10 producers.

To test the latter conclusion in an *in vivo* setting, total and IL-10^−^ CD4^+^ T-cells from CD28SA pretreated Thy1.1 mice (containing 27% and 20% Foxp3^+^ cells, respectively), were labeled with CFSE and transferred into congenic C57BL/6 mice which were treated with CD28SA 24 hours later to further stimulate expansion and IL-10 production. Analysis of lymph node cells after three days showed that mainly Foxp3^+^ cells had proliferated (**Fig. 5D**), and that virtually all IL-10 producers expressed Foxp3. Interestingly, the proportion of IL-10^+^ cells among Tregs was even higher in mice which had received CD4^+^ T-cells depleted of IL-10 producers as compared to those receiving unseparated CD4^+^ T-cells (12.3% vs. 6.32% IL-10^+^ among Foxp3^+^, 4.38% vs. 1.24% IL-10^+^ among CD4^+^), indicating their efficient de novo differentiation from IL-10^−^ precursors (**Fig. 5D**). More than 90% of IL-10 producers in both groups had proliferated, similar to results obtained with transferred naive CD4^+^ T-cells (**Fig. 1E**). Taken together, IL-10 production by Tregs is not a stable phenotype *in vitro* or *in vivo*. Rather, IL-10 production is acquired after clonal expansion and differentiation towards a tissue-seeking phenotype.

### IL-10 producing Tregs are apoptosis-prone

**Figure 6:**
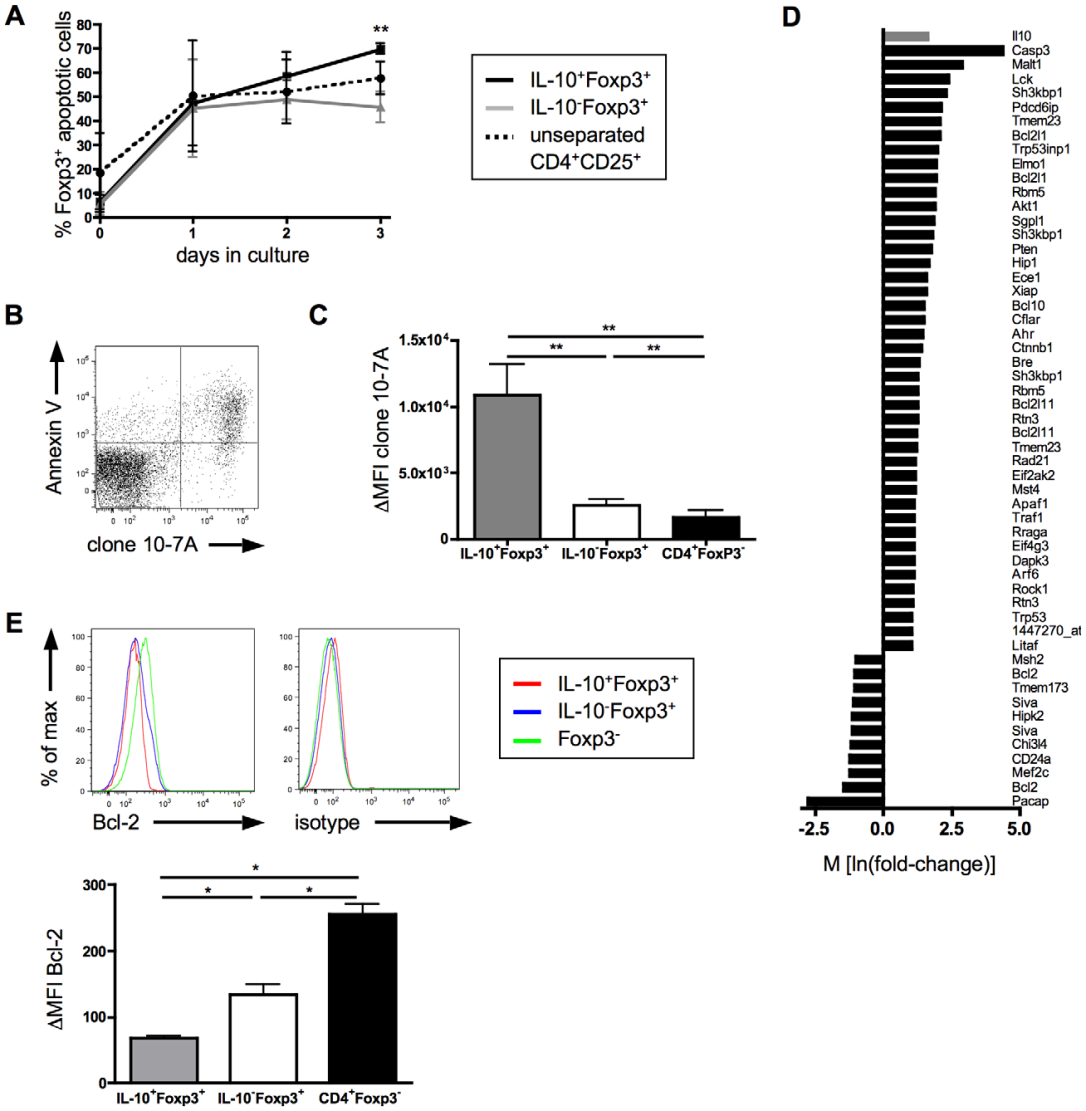
IL-10 producing Tregs are apoptosis prone. (**A**) CD4^+^CD25^+^ Tregs from CD28SA treated DEREG mice were isolated and stimulated over night with anti-CD3, anti-CD28, IL-2. Next day cells were stained for IL-10 and separated into IL-10^+^Foxp3^+^ (black line) and IL-10^−^Foxp3^+^ (grey line) via FACS Sorter and cultured for 1 to 3 days with anti-CD3, anti-CD28, IL-2 and examined for Annexin V and 7AAD positive cells. As control unseparated CD4^+^CD25^+^ T-cells (dashed line) were used. Graphs show means ± SD from 3 individual experiments. ** p<0.005 compared IL-10^+^Foxp3^+^ to IL-10^−^Foxp3^+^ cells. (**B**) Flow cytometric analysis of costaining of Annexin V and clone 10-7A (phospholipids). (**C**) Binding of clone 10-7A given in ΔMFI ( = MFI(marker)−MFI(isotype)) on gated CD4^+^ T-cells 3 days after CD28SA injection dependent on IL-10 and Foxp3 expression. Graphs show means ± SD from 3 or 5 mice assayed individually and results are representative of two independent experiments. ** p<0.005. (**D**) Gene expression data given in M (ln(fold change)) of functional cluster “apoptosis” (DAVID) of sorted IL-10^+^ and IL-10^−^ CD4^+^CD25^+^ Tregs 3 days after stimulation. Transcripts selected for genes that showed ≥2-fold change and an adjusted p-value<0.001 (n = 2). (**E**) Bcl-2 expression of gated CD4^+^ T-cells 3 days after CD28SA injection dependent on IL-10 and Foxp3 expression. * p<0.05. Results are representative of three independent experiments with 3–4 mice.

The continuous decline of viable cells recovered from optimally stimulated cultures of IL-10-producing Tregs (**Fig. 5A**) suggested that this high degree of functional activation is accompanied by increased sensitivity to apoptosis, thereby self-limiting immunosuppression. We therefore followed purified IL-10 producing and non-producing Tregs (the same cultures as analyzed in **Fig. 5A**) with regard to the expression of an apoptotic phenotype over time (**Fig. 6A**). Indeed, we observed a continuous increase of Annexin V and 7AAD positive cells, whereas their frequency stabilized in cultures derived from IL-10^−^ Tregs after the first day of culture (**Fig. 6A**).

To further characterize pre-apoptotic cells, we developed a novel mAb, 10-7A, which binds to phospholipids (phosphatidylethanolamine, phosphatidylserine and cardiolipin) exposed on such cells at the outer leaflet of the plasma membrane (**Fig. S2A, B**). As shown in **Figure 6B**, this mAb detects most Annexin V reactive cells, but provides superior resolution. In fact, *ex vivo* staining of CD28SA activated lymph node cells for exposed phospholipids revealed that IL-10 producing Tregs express much higher levels as compared to IL-10 non-producers and conventional CD4^+^ T-cells (**Fig. 6C**).

Finally, the hypothesis that IL-10 secreting Tregs are more apoptosis-prone than their IL-10-negative counterparts was further tested by gene expression analysis. Several pro-apoptotic genes like Caspase 3, Pten, Bcl2l11 (Bim), Rock1 or Apaf1 were upregulated in IL-10 producing compared to IL-10 negative Tregs, whereas the anti-apoptotic gene Bcl-2 was downregulated (**Fig. 6D** **and S3A**), a finding which was confirmed by flow cytometric measurement at the protein level (**Fig. 6E**).

## Discussion

Here we report that Tregs polyclonally activated with CD28SA *in vivo* not only dramatically expand in numbers, but also initiate IL-10 production and upregulate effector molecules and homing receptors guiding them to inflamed tissue. Furthermore, suppression was self-limiting as IL-10 production was followed by apoptosis of the effector Tregs.

Previous work by others has shown that *in vitro*, a fraction of Tregs acquires competence for IL-10 production after several rounds of cell division driven by TCR plus IL-2 stimulation [3], [4]. Here, we show that this conversion also occurs, and with an even higher efficiency, during polyclonal Treg stimulation *in vivo*, where it also depends on previous cycling. Thus, proliferating Tregs in CD28SA stimulated mice initially contained only very few (<2%) IL-10 producers, but this proportion dramatically increased to about 25% by day 3, with peak frequencies in Tregs having undergone 5–7 cell divisions (**Fig. 1**). Furthermore, FACS-sorted non-IL-10 producing Tregs restimulated *in vivo* via CD28SA initiate IL-10 secretion (**Fig. 5D**). By transfer experiments we could demonstrate that conversion of Tconv into Foxp3^+^ and/or IL-10^+^ cells does not contribute to the enriched numbers of IL-10^+^ Foxp3^+^ cells in our system (**Fig. 1D**). Mechanistically, the *in vitro* and *in vivo* observations on IL-10 induction in Tregs are most likely equivalent because CD28SA amplifies weak TCR signals [24], and IL-2 is provided by concomitant stimulation of conventional CD4^+^ T-cells [19]. Treg conversion from an IL-10 incompetent to an IL-10 competent phenotype was also observed *in vivo* using Foxp3/IL-10 reporter mice under steady-state conditions [7]. Since Tregs undergo continuous homeostatic turnover, this is not in contrast to our conclusion that cell division precedes expression of IL-10.

Both in resting [7] and in CD28SA stimulated mice (**Fig. 2**), IL-10 competent Tregs have features of activated or memory T-cells (CD44^high^, CD45RB^low^, GITR^high^, ICOS^high^). Importantly, IL-10 producing Tregs from CD28SA stimulated mice were particularly high in the expression of regulatory effector molecules which was most prominent for CTLA-4 with a 2.5-fold higher expression than on their IL-10 negative counterparts, and a 20-fold upregulation above the constitutive level on resting Tregs (**Fig. 2**). Recently, the importance of this receptor for Treg function was underscored by its ability to remove costimulatory ligands (CD80, CD86) from APCs by trans-endocytosis [27].

*In vivo* CD28SA activated Tregs demonstrated dramatically enhanced suppressor activity *in vitro* ([19], **Fig. 3**) and also *in vivo* [19], [28]. Importantly, we observed a particularly high, while IL-10 independent, suppressive activity within the IL-10 producing subset of *in vivo* activated Tregs, correlating with their high level of regulatory cell surface receptors.

Tissue-specific homing receptors were previously shown to be upregulated on effector/memory-like Tregs [12], [29]. In agreement with these reports, CD28SA stimulation downregulated the lymphoid homing-receptor CCR7 [13], but upregulated the inflammation-seeking receptor CCR5 and CCR6 [13], [30], [31] on Tregs. In accordance with the view that polyclonally (CD28SA) activated IL-10 producers are a correlate, at the population level, of the few Tregs in unmanipulated mice producing IL-10 in response to (self)antigens, these also expressed tissue-homing receptors (CD103, CCR5, CCR6) consistent with their preferential recruitment to inflamed sites (**Fig. 4A** and data no shown).

A key finding of the present study is that after clonal expansion and acquisition of IL-10 competence and tissue-homing capability, most of these highly activated Tregs undergo apoptosis, and most of the remaining cells lose the capacity to produce IL-10, ensuring termination of immunosuppression after clearance of an infection. The preferential apoptosis of IL-10 producing Tregs (**Fig. 1E** and **6A**) is in line with the well-known link between extensive proliferation and apoptosis [32]. Activation-induced cell death depending in part on death receptor CD95 and its ligand CD95L is one mechanism that controls the pool of conventional CD4^+^ T-cells, but data regarding the sensitivity to CD95-mediated apoptosis of Tregs are inconsistent [33], [34]. For human Tregs it was shown that effector/memory Tregs are highly sensitive to apoptosis whereas naïve/resting Tregs are more resistant [34]. In line with this, we and others observed downregulation of the anti-apoptotic protein Bcl-2 (**Fig. 6E** and [35]) and upregulation of pro-apoptotic proteins like Bim or Caspase 3 in effector/memory-like IL-10 producing Tregs after activation. However, upregulation of CD95L was not accompanied by upregulation of CD95 (data not shown) after CD28SA stimulation. Furthermore it is unlikely that IL-10 itself, which can induce apoptosis in several cell types like mast cells, macrophages and CD4^+^ T cells from SLE patients [36], [37] is responsible for the preferential apoptosis of IL-10 producing Tregs since *in vivo* CD28SA activated IL-10 producing and non-producing Tregs express comparable and low levels of IL-10R (**Fig. S3B**). Thus, it is not clear yet which mechanism(s) primarily drive(s) apoptosis of IL-10 producing Tregs.

Taken together, our data connect clonal expansion, functional differentiation as highlighted by competence for IL-10 production, migration to inflamed sites, and programmed cell death within a polyclonal wave of Treg activation. This sequence of events correlates well with the striking efficacy of CD28SA treatment in a broad spectrum of murine immune pathologies, and with the return of the immune system to normality once Treg levels have subsided in these models. While the unexpected cytokine release syndrome induced by a human CD28SA [38], now traced back to the stimulation of effector memory CD4 T-cells [39], [40], interrupted the development of this strategy in humans, transient polyclonal Treg activation remains an attractive, and broadly applicable approach for the future.

## Materials and Methods

### Ethics statement

All experiments were performed according to the German regulations for animal experimentation and approved by the Government of Lower Franconia as the responsible authority (Permit Numbers 55.2-2531.01-64/07 and -64/11).

### Mice

C57BL/6 (Harlan Winkelmann, Borchen, Germany), congenic Thy1.1^+^C57BL/6 mice (Jackson Laboratories) and DEREG mice generously provided by Tim Sparwasser [26] were bred in the institute's barrier-facility and used between 6–12 weeks of age.

### *In vivo* expansion of T-cells by superagonistic anti-CD28 mAb

CD28SA (D665) [25] was bioreactor-produced in a low-endotoxin format by Exbio, Prague, Czech Republic, Invivo Biotech, Henningsdorf, Germany, or Serotec, Oxford, UK. PBS was used as negative control. 200 µg per mouse was injected intraperitoneally.

### Flow cytometry

Fluorochrome-labeled or biotinylated Abs to the following proteins were used: CD4 (RM4-5), CD25 (7D4), CD103 (M290), CD62L (MEL-14), CD44 (Ly-24), CD45RB (16A), CD90.1 (OX-7), GITR (DTA-1), ICOS (7E.17G9), CCR5 (C34-3448), CCR6 (140706), Bcl-2 (3FM) and IL-10 (JES5-16E3) from BD Biosciences and LAP (TW7-16B4), IL-10R (1B1.3a) from BioLegend. For intracellular IL-10 staining cells were restimulated with PMA (0.1 µg/ml, Sigma-Aldrich), Ionomycin (1 µg/ml, Sigma-Aldrich) and Golgistop (BD) for 4 hours, washed, fixed with Fix/Perm Buffer from eBioscience and permeabilized with Perm Buffer (BD). Foxp3 (FJK-16s), CCR7 (4B12), PD-1 (J43), CTLA-4 (UC10.4B9), CD39 (24DMS1) and staining reagents from eBioscience were used according to the manufacturer's instructions. Acquisition was performed on a FACSCalibur™ or BD™ LSR II and data were analyzed using FlowJo software (TreeStar Inc, Ashland, OR, USA). 10-7A is an IgM antibody generated from mice immunized with a plasma membrane fraction isolated from CD28SA activated rat Tregs. It was shown by ELISA (modified after [41]) that it is specific for phospholipids (phosphatidylethanolamine, phosphatidylserine and cardiolipin). As secondary antibody we used PE labeled anti-IgM (R6-60.2, BD).

### Cell isolation and cell culture

Single-cell suspensions of lymph nodes were stained with a cocktail of biotin-labeled antibodies (BD), followed by incubation with Streptavidin MicroBeads. CD4^+^ T-cells were prepared by negative selection using the MACS separation system (Miltenyi Biotech). CD25^+^ and CD25^−^ CD4^+^ T-cells were separated using biotin-labeled anti-CD25 mAb (7D4) and Streptavidin MicroBeads. IL-10 producing cells were stained with IL-10 Secretion Assay (Cell Enrichment and Detection Kit) from Miltenyi Biotec according to manufacturer's instructions and sorted for IL-10 positive and negative cells via Cellsorter Vantage Diva. Cells were cultured with anti-CD3 (145-2C11, 2 µg/ml, BD Biosciences), anti-CD28 (E18, 5 µg/ml, Exbio, Prague, Czech Republic) and recombinant human IL-2 (200 U/ml, Novartis, Basel, CH) for up to three days. For detection of apoptosis cells were stained with Annexin V and 7AAD (BD Biosciences).

### Adoptive transfer

Purified CD4^+^ T-cells or purified IL-10^−^ CD4^+^ T-cells of untreated or CD28SA stimulated Thy1.1 mice (day 3) were labeled with 10 µM CFSE (Invitrogen) at RT for 5 min. A total of 1.0×10^7^ cells in PBS were transferred i.v. one day before CD28SA stimulation. Average cell division numbers (acd) were calculated as Σ(% of cells in generation (n)×n)/100, (n = number of generation). For conversion experiments purified CD4^+^ T-cells of DEREG mice were FACS sorted for Foxp3^−^(GFP^−^) cells. A total of 7.0×10^6^ cells in PBS were transferred i.v. into congenic Thy1.1 mice one day before CD28SA stimulation.

### *In vitro* suppression assay

Isolated CD4^+^CD25^−^ responder T-cells (5×10^4^) were CFSE labeled and cultured for 3 days in U-bottomed 96-well plates with various dilutions of purified IL-10^+^, IL-10^−^ or CD4^+^CD25^+^/Foxp3^+^(GFP^+^) Tregs in the presence of soluble anti-CD3 (1 µg/ml), irradiated splenic APC (20 Gy; 2×10^5^) and where mentioned anti-IL-10R (1B1,2, 10 µg/ml). Proliferation was assayed by CFSE dilution.

### Microarray analysis

Total RNA was extracted from isolated IL-10^+^ and IL-10^−^ CD4^+^CD25^+^ T-cells from C57BL/6 mice by TRIZOL (Invitrogen) method. Expression profiling analysis of RNA from IL10^+^ and IL-10^−^ Tregs was performed on GeneChip® Mouse Genome 430 2.0 (Affymetrix, Santa Clara, CA). RNA quality was checked using a BioAnalyzer (Agilent). RNA integrity numbers (RIN) of the RNAs were between 9.4 and 9.6. Reverse transcription, second-strand synthesis and cleanup of double-stranded cDNA were performed according to the Affymetrix protocols (One-Cycle cDNA synthesis Kit, Affymetrix, Santa Clara, CA) starting from 2 µg of total RNA. Data were analyzed using different R packages from the Bioconductor project (www.bioconductor.org). Resulting signal intensities were normalized by variance stabilization [42]. The quality of all data sets was tested by density plot and RNA degradation plot. Statistical analysis to select differentially expressed genes was performed using the LIMMA (Linear Models for Microarray Analysis) package [43]. To find transcripts that were different between two samples, we selected for genes that showed a fold change of at least 2-fold to maximal 79-fold and an adjusted p-value<0.001. To evaluate functional categories and to map the selected genes to pathways DAVID (http://david.abcc.ncifcrf.gov/home.jsp) database was used. The microarray data are available in the Gene Expression Omnibus (GEO) database (http://www.ncbi.nlm.nih.gov/gds) under the accession number GSE41492.

### Sponge implantation

Polyester-polyurethane sponges (5×5 mm^2^) (Vitaform Ltd, Manchester, UK) containing heatkilled *Listeria monocytogenes* were implanted s.c. in C57BL/6 mice [44]. After 4 days mice were treated with 200 µg CD28SA or PBS i.p.. On day 7 sponges were recovered, cells were washed out several times with BSS/BSA and stained for Foxp3 and IL-10 via IL-10 Secretion Assay.

### Statistical analysis

Data are presented as mean ± SD. Statistical significance was analyzed by unpaired *t*-test or Mann-Whitney U test using GraphPad Prism Software. Values of p<0.05 were considered to be statistically significant.

## Supporting Information

Figure S1 **Expression of migration related receptors of Tregs after CD28SA injection.** Percent of migration related receptor positive cells in unstimulated Foxp3^+^ and Foxp3^−^ CD4^+^ T-cells and 3 days after CD28SA stimulation in IL-10^+^ Foxp3^+^ (grey bar), IL-10^−^Foxp3^+^ (white bar) and Foxp3^−^ (black bar) CD4^+^ T-cells. Graphs show means ± SD from 3–4 mice assayed individually and results are representative of two independent experiments. * p<0.05, ns: no significant difference.
(TIF)

Figure S2 **Characterisation of clone 10-7A.** (**A**) Surface staining of isolated *in vivo* CD28SA activated rat T-cells after 48 h culture in medium+IL-2 with clone 10-7A and CD25. (**B**) ELISA, which identified phosphatidylethanolamine, phosphatidylserine and cardiolipin as target antigens for clone 10-7A mAb.
(TIF)

Figure S3 **Expression of IL-10R and genes associated with apoptosis.** (**A**) Gene expression data of functional cluster “apoptosis” (DAVID) of sorted IL-10^+^ and IL-10^−^ CD4^+^CD25^+^ Tregs 3 days after stimulation. Ratio: gene expression level of IL-10^+^ to IL-10^−^ CD4^+^CD25^+^ cells, M: ln(fold change). Transcripts selected for genes that showed ≥2-fold change and an adjusted p-value<0.001 (n = 2). (**B**) IL-10R expression of lymph node cells 3 days after CD28SA injection dependent on CD4, IL-10 and Foxp3 expression. * p<0.05. Results are representative of two independent experiments with 4–5 mice.
(TIF)
