# Supplementary figures and images for: Sequential Induction of Effector Function, Tissue Migration and Cell Death during Polyclonal Activation of Mouse Regulatory T-Cells
Source: PLoS One. 2012 Nov 30;7(11):e50080. doi: 10.1371/journal.pone.0050080 (PMC3511437; doi:10.1371/journal.pone.0050080)

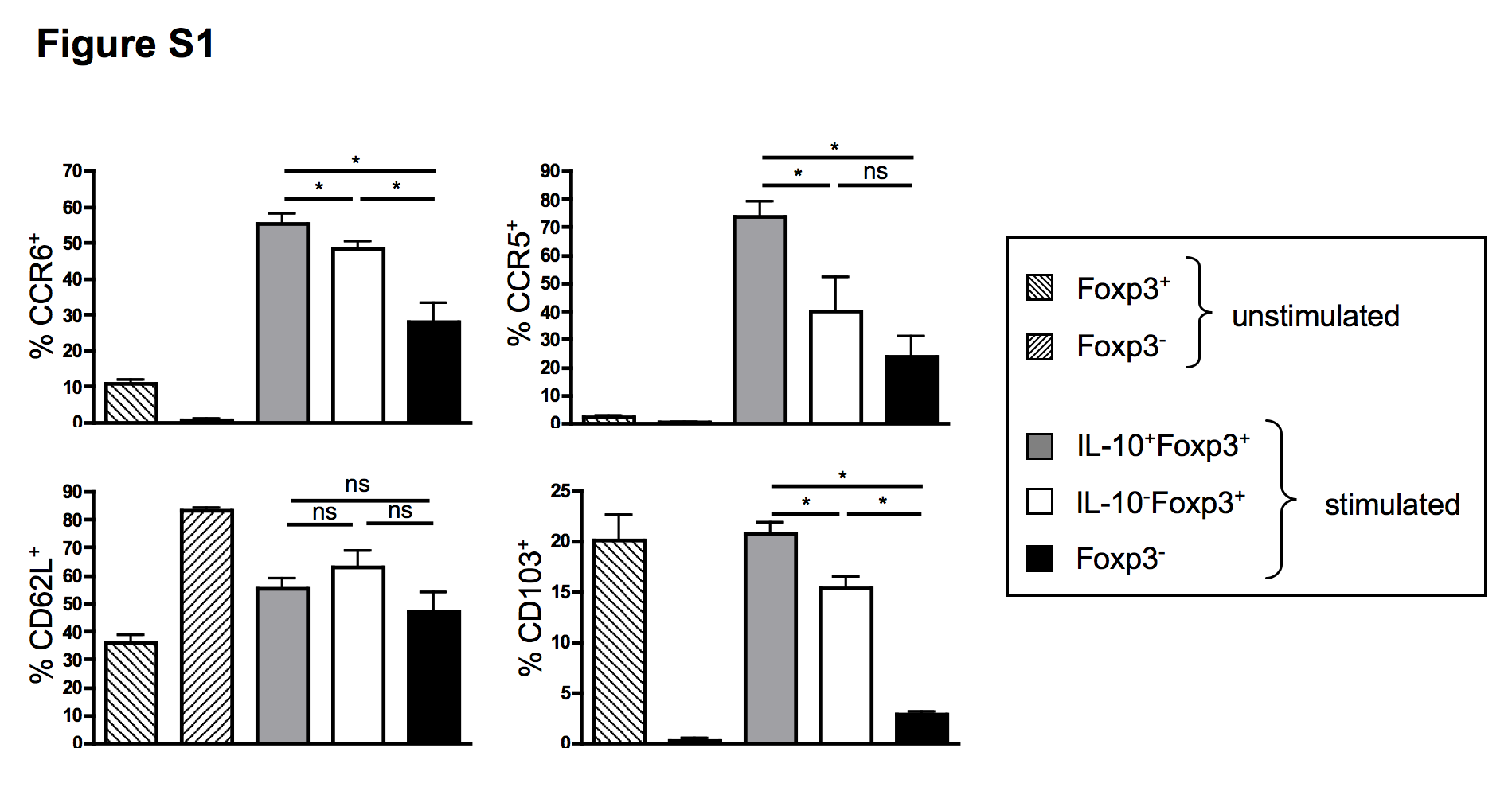

Supplement: Figure S1 — Expression of migration related receptors of Tregs after CD28SA injection. Percent of migration related receptor positive cells in unstimulated Foxp3+ and Foxp3− CD4+ T-cells and 3 days after CD28SA stimulation in IL-10+ Foxp3+ (grey bar), IL-10−Foxp3+ (white bar) and Foxp3− (black bar) CD4+ T-cells. Graphs show means ± SD from 3–4 mice assayed individually and results are representative of two independent experiments. * p<0.05, ns: no significant difference. (TIF) [file pone.0050080.s001.tif]

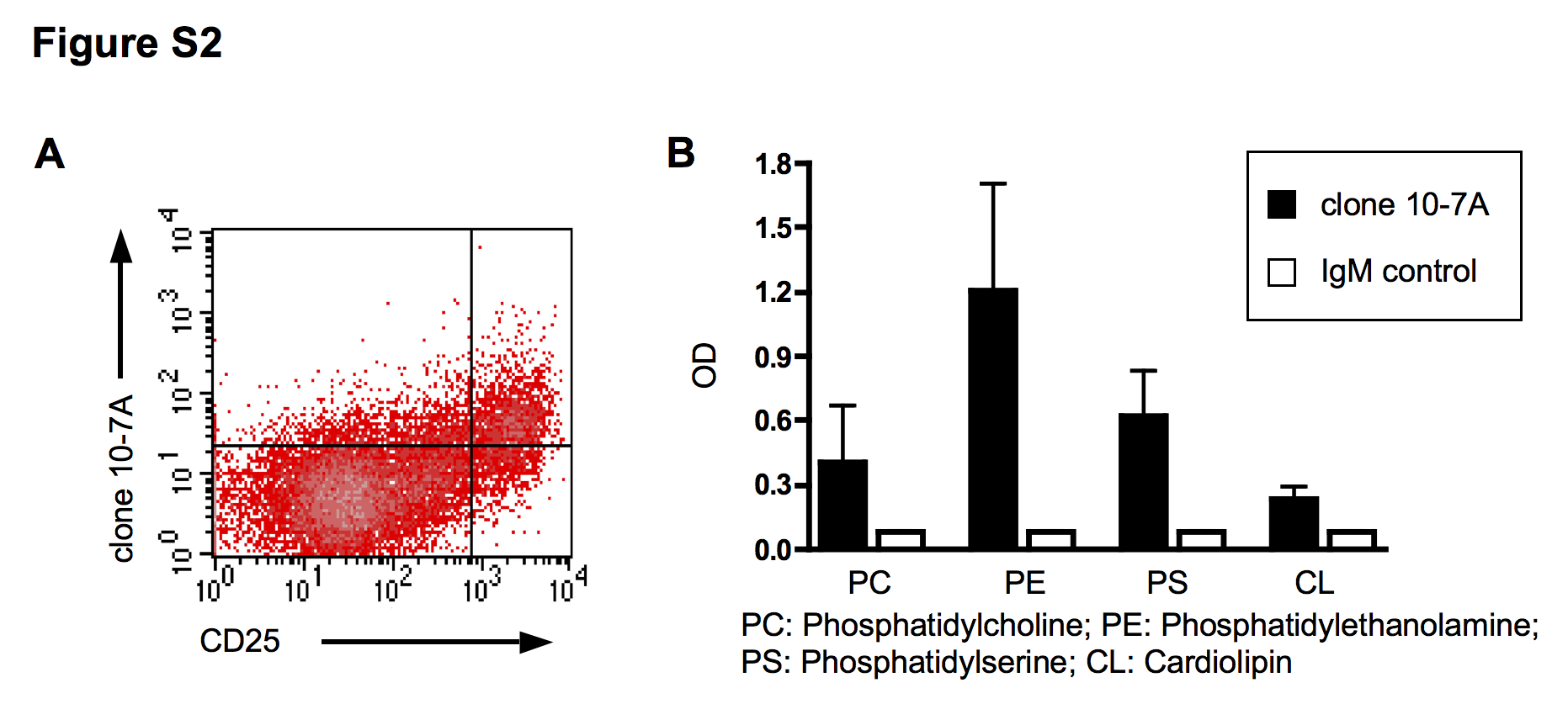

Supplement: Figure S2 — Characterisation of clone 10-7A. (A) Surface staining of isolated in vivo CD28SA activated rat T-cells after 48 h culture in medium+IL-2 with clone 10-7A and CD25. (B) ELISA, which identified phosphatidylethanolamine, phosphatidylserine and cardiolipin as target antigens for clone 10-7A mAb. (TIF) [file pone.0050080.s002.tif]

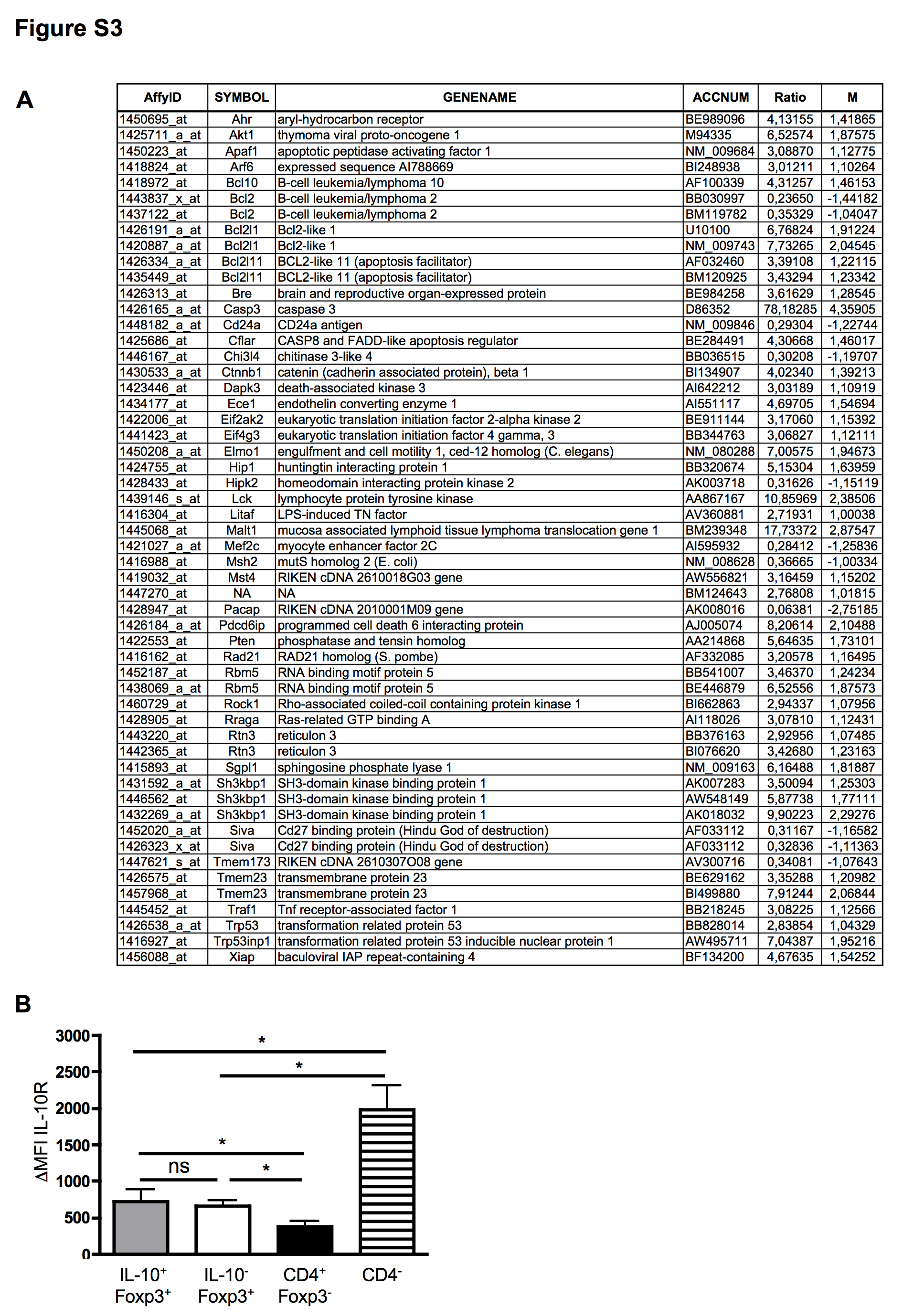

Supplement: Figure S3 — Expression of IL-10R and genes associated with apoptosis. (A) Gene expression data of functional cluster “apoptosis” (DAVID) of sorted IL-10+ and IL-10− CD4+CD25+ Tregs 3 days after stimulation. Ratio: gene expression level of IL-10+ to IL-10− CD4+CD25+ cells, M: ln(fold change). Transcripts selected for genes that showed ≥2-fold change and an adjusted p-value<0.001 (n = 2). (B) IL-10R expression of lymph node cells 3 days after CD28SA injection dependent on CD4, IL-10 and Foxp3 expression. * p<0.05. Results are representative of two independent experiments with 4–5 mice. (TIF) [file pone.0050080.s003.tif]
